# DNA Copy Number Aberrations and Expression of ABC Transporter Genes in Breast Tumour: Correlation with the Effect of Neoadjuvant Chemotherapy and Prognosis of the Disease

**DOI:** 10.3390/pharmaceutics14050948

**Published:** 2022-04-27

**Authors:** Matvey M. Tsyganov, Marina K. Ibragimova, Kseniya A. Gaptulbarova, Irina A. Tsydenova, Daria S. Dolgasheva, Evgeniy Y. Garbukov, Anastasia A. Frolova, Elena M. Slonimskaya, Nikolai V. Litvyakov

**Affiliations:** 1 Cancer Research Institute, Tomsk National Research Medical Center, Russian Academy of Sciences, 5, Kooperativny Street, 634050 Tomsk, Russia; imk1805@yandex.ru (M.K.I.); xenia.gaptulbarova@yandex.ru (K.A.G.); tsydenova422@gmail.com (I.A.T.); normikus.18.97@gmail.com (D.S.D.); jrmaximum@rambler.ru (E.Y.G.); anastasiya10152@gmail.com (A.A.F.); slonimskaya@rambler.ru (E.M.S.); nvlitv72@yandex.ru (N.V.L.); 2 Biological Institute, Tomsk State University, 36 Lenin Ave., 634050 Tomsk, Russia

**Keywords:** ABC transporters, gene expression, DNA copy number aberrations, neoadjuvant chemotherapy, prognosis

## Abstract

One of the important reasons for the ineffectiveness of chemotherapy in breast cancer (BC) is considered to be the formation of a multidrug resistance phenotype in tumour cells, which is caused by the expression of energy-dependent ABC transporters. The aim of this work was to assess chromosomal aberrations and the level of transcripts of all 49 known ABC transporter genes in breast tumours. Materials and Methods. The study included 129 patients with breast cancer. A microarray study of all tumour samples was carried out on microchips. Results. This study established that the presence of a deletion in genes *ABCB1, ABCB4, ABCB8, ABCC7, ABCC11, ABCC12, ABCF2,* and *ABCG4* is associated with an objective response to treatment (*p* ≤ 0.05). A decrease in the expression of genes was associated with a good response to chemotherapy, whereas an increase in expression caused the progression and stabilization of the tumour. Analysis of metastatic-free survival rates showed that the presence of *ABCB1/4* and *ABCC1/6* deletions was associated with 100% survival (log-rank test *p* = 0.01 and *p* = 0.03). Conclusions. The study showed that the aberrant state of ABC transporter genes, as well as a decrease in the expression of these genes, is a predictor of the effectiveness of therapeutic treatment and a potential prognostic marker of metastatic survival.

## 1. Introduction

Systemic chemotherapy is an important component of the complex treatment of patients with breast cancer (BC); various chemotherapy regimens based on anthracycline drugs, 5-fluorouracil, platinum drugs, herbal drugs, etc., are used as standard treatments [1]. An important reason for the chemotherapy inefficiency of cancers in various locations in the body, including breast cancer, is the formation of a phenotype of multiple drug resistance (MDR) before and during treatment [2]. At the same time, the mechanisms of action mediating the development of MDR appear to be quite complicated; these mechanisms include but are not limited to increased extracellular drug efflux, decreased intracellular drug uptake, alterations in drug metabolism, mutations of drug targets, and inactivation of death signalling pathways [3]. These mechanisms can generally be divided into two broad groups: intrinsic and acquired. Intrinsic resistance occurs when MDR is naturally expressed in tumours, whereas acquired resistance occurs when MDR develops as a result of chemotherapy. Thus, the effectiveness of chemotherapeutic agents might be limited by intrinsic resistance before treatment begins and can worsen during treatment due to acquired resistance [4].

The efficiency of chemotherapy for breast cancer patients is limited by individual drug sensitivity, targets for therapy, and cancer-cell-nonspecific resistance [5]. It is well known that xenobiotics, including anticancer drugs, are metabolized and transported in the body through various mechanisms, particularly the process of biotransformation [6]. The drug transport process depends on the expression of a group of energy-dependent ABC transporters (ATP-binding cassette transporters), which play a decisive role in the development of multidrug resistance in tumour cells [7,8]. The primary members of the ABC transporter family leading to doxorubicin resistance in cancer cells are *ABCBs*, *ABCCs*, and *ABCG2* [9]. *ABCB1, ABCG2, ABCC1,* and *ABCC2* transporter genes are highly polymorphic and may influence the excretion and elimination of docetaxel and further affect its pharmacokinetics and pharmacodynamics [10]. In addition, in vitro studies in MDA-MB-231 cells showed that the suppression of *ABCG2* expression increases the effectiveness of doxorubicin [11,12]. The role of *ABCC5* in the efflux of pemetrexed in MCF-7 cells was established, and *ABCC5* expression was inversely correlated with sensitivity to this drug (r = 0.741; *p*  < 0.001) in breast tumour cells obtained from patients [13]. Additionally, in vitro studies have shown the role of transporter genes in resistance to cisplatin [14]. Importantly several studies have reported that ABC transporters influence the formation of multiple drug resistance during tumour progression, invasion, and metastasis [15,16]. Our previous study showed that changes in the expression levels of several ABC genes (*ABCB1, ABCC1, ABCC2, ABCC5, ABCG1,* and *ABCG2*) during neoadjuvant chemotherapy (NAC) were associated with the response to this therapy in breast cancer patients [2]. A decrease in the expression of these genes determines the formation of an objective response to treatment (complete and partial regression), whereas an increase indicates a lack of response (progression and stabilization of the tumour).

Additional studies have established that the factors involved in the regulation of changes in the expression of ABC genes could be various aberrant states, in particular, DNA copy number aberration (CNA), which is a type of structural variation, specifically, a type of amplification or deletion event that affects a considerable number of base pairs [17]. This type of disruption can be a good prognostic marker. Thus, the increased frequency of DNA copy number aberrations in the 1q21, 8q, 14q24, Xp11.2-p21, 6q16, and 6q21-q22 regions of chromosomes is associated with a low level of disease-free and overall survival [18]. Deletions of multiple drug resistance gene loci have been found to lead to decreased expression in response to chemotherapy and are associated with a good response to preoperative chemotherapy [19,20]. Methylation of the promoter regions of ABC genes also affects their own expression. ABC genes in tumours of various localizations often have hypomethylated promoters, which may cause their overexpression [21,22].

Unfortunately, in the vast majority of cases, the study of ABC transporters in clinical material is limited to 5–10 genes of the 49 in this family, which is important for the formation of multiple drug resistance. However, the significance of other genes from the ABC transporter family also cannot be completely excluded.

A recent study showed that reduced expression of *ABCC4, ABCC10, ABCD3, ABCE1, ABCF1, ABCF2,* and *ABCF3* was associated with superior chemotherapy response and a long time to progression in ovarian cancer patients. At the same time, a decrease in the activity of *ABCB11* and an increase in the activity of *ABCB1* and *ABCG2* were associated with less sensitivity to chemotherapy [23]. There are also studies on the expression profile of all ABC transporter genes; in 2013, the genes *ABCA5/6/8/9/10, ABCB1/5/11, ABCC6/9, ABCD2/4, ABCG5*, and *ABCG8* were shown to be significantly downregulated, and the genes *ABCA2/3/7/12, ABCB2/3/8/9/10, ABCC1/4/5/10/11/12, ABCD1/3, ABCE1, ABCF1/2/3*, and *ABCG1* were shown to be upregulated in post-treatment tumours compared with non-neoplastic tissues. *ABCA12*, *ABCA13*, and *ABCD2* have been associated with the response to neoadjuvant chemotherapy in patients with breast cancer after treatment [24]. However, these studies are limited in that they only considered the expression of these genes in the surgical material and do not provide data on changes in gene expression during treatment or the relationship of those changes with survival and efficacy of neoadjuvant chemotherapy.

Thus, the aim of this work was to assess chromosomal aberrations and the transcript level of all 49 known ABC transporter genes in breast tumours before and after treatment, as well as their predictive and prognostic potential.

## 2. Materials and Methods

### Patients and Treatment

This study included 129 breast cancer patients of stages IIA–IIIB (T_1–4_N_0–3_M_0_) with a morphologically verified diagnosis, aged 28–68, with an average age of 47.43 ± 0.78 years (mean ± SE) (Table 1). All patients received treatment from 2006 to 2020 at the Research Institute of Oncology, Tomsk National Research Medical Center of the Russian Academy of Sciences (Tomsk, Russia). The study was conducted according to the ethical principles suggested in the Declaration of Helsinki (fixed in 2013) [25] and approved by the Ethical Committee of the Cancer Research Institute. Signed informed consent was obtained from all participants. An immunohistochemical study was performed prior to treatment to determine the molecular subtype of the tumour. The luminal B subtype of breast cancer was determined by ER+, PR+ or −, Ki67 > 30%, and all patients with the luminal B subtype were HER2-negative. HER2 testing was performed in accordance with the American Society of Clinical Oncology/College of American Pathologists Guideline 2007 Recommendation [26]. The HER2-positive molecular subtype was determined by positive expression of HER2 (+++) or HER2 gene amplification by the FISH method (HER2 (++)). Patients with the triple-negative molecular subtype did not express the oestrogen, progesterone, or HER2 receptor molecule.

In accordance with the consensus conference on neoadjuvant chemotherapy in carcinoma of the breast, 26–28 April 2003, Philadelphia, Pennsylvania [25], all patients received 4–8 courses of neoadjuvant chemotherapy: AC (adriamycin 50 mg/m^2^ and cyclophosphamide 600 mg/m^2^ every 3 weeks), AT (adriamycin 50 mg/m^2^ and taxotere 75 mg/m^2^), ACT (adriamycin 50 mg/m^2^, cyclophosphamide 600 mg/m^2^, and taxotere 75 mg/m^2^), CAX (cyclophosphamide 100 mg/m^2^ intramuscularly, adriamycin 30 mg/m^2^ intravenously, and Xeloda 1200 mg/m^2^ orally), CP (cyclophosphamide 1080 mg/m^2^ and cisplatin 135 mg) or monotherapy taxotere (100 mg/m^2^, 1 h infusion per day). The effect of chemotherapy was determined according to the criteria of the World Health Organization. A complete response (CR) was defined as complete disappearance of the primary tumour and lymph node metastasis. A partial response (PR) was defined as a tumour reduction >50%, and stabilization (ST) was defined as a tumour reduction ≤50% or a tumour size increase of <25%. Progression (P) was described as an increase of >25% in tumour size. After 3–5 weeks of NAC, all patients underwent radical or subcutaneous mastectomy, radical resection, sectoral resection with axillary lymphadenectomy, or other types of organ-preserving surgery; then, the patients underwent radiation and/or hormonal or targeted therapy (Herceptin for HER2 + status) according to indications. During the entire period, the patients were monitored dynamically. The median follow-up time was 44 months (44 ± 2.79).

Tumour biopsies (~10 mm^3^) were taken from all patients before (~10 mm^3^) and after (~60–70 mm^3^) neoadjuvant treatment (3–5 weeks after the last course of chemotherapy). All tumour samples were placed in RNAlater solution (Sigma, St. Louis, MO, USA) and stored at −80 °C (after 24 h incubation at +4 °C) for subsequent RNA/DNA extraction.

DNA extraction. DNA was extracted from 129 tumour biopsy samples with a QIAamp DNA mini kit (Qiagen, Germany). DNA quality and concentration were assessed on a fluorometer Qubit 4.0 (Agilent Technologies, Santa Clara, CA, USA), from 50 to 250 ng/mcl. DNA integrity was assessed by capillary electrophoresis on a TapeStation 4150 device (Agilent Technologies, Santa Clara, CA, USA); DNA fragments were over 60 kbp.

Microarrays assay. Microarray analysis was performed on high-density microarrays (DNA chips) from Affymetrix (USA), and a CytoScan^TM^ HD Array was used to evaluate the CNA performance, which contains 1 million 900 thousand non-polymorphic markers for the analysis of copy number aberrations. Sample preparation, hybridization, and scanning procedures were performed in accordance with the manufacturer’s protocol for the Affymetrix GeneChip^®^ Scanner 3000 7G system (Affymetrix, Santa Clara, CA, USA). Chromosome Analysis Suite 4.1 (Affymetrix, Santa Clara, CA, USA) was used for statistics. As a result of the analysis, amplifications (gain) and deletions (loss) of the ABC genes were determined.

*RNA extraction.* RNA was extracted from 39 paired samples before and after NAC with an RNeasy Plus mini kit (Qiagen, Germany). RNA quality and concentration were evaluated on a fluorometer Qubit 4.0 (Agilent Technologies, Santa Clara, CA, USA), from 20 to 100 ng/mcl. RNA integrity was evaluated with automated electrophoresis using a TapeStation 4150 (Agilent Technologies, Santa Clara, CA, USA). RIN was 6.4–8.1.

*Study of ABC-transporter gene expression.* Analysis of the expression of the 49 ABC transporter family genes was carried out on a Clariom™ S Assay microarray platform (Affymetrix, Santa Clara, CA, USA). Such microarrays allow for the analysis of more than 20 thousand of the main human genes. Sample preparation, hybridization, and scanning procedures were performed in accordance with the manufacturer’s protocol for the Affymetrix GeneChip^®^ Scanner 3000 7G system (Affymetrix, Santa Clara, CA, USA). Microchip data were analysed using Transcriptome Analysis Console (TAC) software 4.0. The expression level for each patient was expressed as log base two. Expression data normalization was carried out as described in [27].

*Statistics*. Statistical data processing was carried out using the application package Statistica 8.0 (StatSoft Inc., Palo Alto, CA, USA). The Shapiro–Wilk test was used to check the normality of the sample. For each sample, the mean value and the mean square error were calculated. The nonparametric Wilcoxon–Mann–Whitney test was used to test the hypothesis about the significance of differences between the study groups. Bonferroni’s correction was used to correct the significance levels for multiple comparisons, calculated as a ratio of 0.05/49, where 49 is the number of analysed genes. The *p* level was statistically significant, adjusted for multiple comparisons, at 0.001. Survival curves were constructed by the Kaplan–Meier method and the log-rank test for the analysis of overall and metastatic-free survival. The chi-square test was used to assess the differences in the frequencies of decrease and increase in gene expression in groups with an objective response to NAC and no response to NAC (http://vassarstats.net/index.html, (accessed on 25 April 2022)).

## 3. Results

### 3.1. Copy Number Aberration of the ABC Transporter Genes

In the first stage of the research, we evaluated the frequency of chromosomal aberrations at ABC gene locations (Supplementary Materials, Table S1) in tumours before treatment. The deletion frequencies for these genes, from highest to lowest, were as follows: *ABCG4* (47.3%, 61/129 cases); *ABCC11* (44.2%, 57/129 cases); *ABCC12* (44.2%, 57/129 cases); *ABCD4* (39.5%, 51/129 cases); *ABCC4* (33.3%, 43/129 cases); *ABCC2* (31.8%, 41/129 cases); and *ABCA7* (31.0%, 40/129 cases). At the same time, a high frequency of amplifications was established for the *ABCA* gene family: *ABCB10* (58.9%, 76/129 cases); *ABCA6* (38.8%, 50/129 cases); *ABCA8* (38.8%, 50/129 cases); *ABCA9* (38.8%, 50/129 cases); *ABCA5* (32.6%, 42/129 cases); and *ABCA10* (32.6%, 42/129 cases).

In the second stage, we analysed the connection between NAC efficiency and clinical and pathological parameters (age, menstrual status, histological type, tumour size, lymphogenous metastasis, and histological form). None of those parameters had a significant impact on the NAC effect (data not shown) or the prognosis of the disease. In addition, metastatic-free survival did not differ by molecular subtype. Next, we analysed the predictive significance of the presence of chromosomal aberrations in ABC genes before treatment and assessed their relationship with the effect of neoadjuvant chemotherapy. Table 2 shows the CNA frequency in the group with an objective response to treatment (complete and partial regression) and without response (stabilization and progression of the tumour).

Table 2 shows that the CNA frequency (Gain and Loss) in the first group of patients reached 69%, whereas in the group without response, it did not exceed 40%. In particular, the deletion frequency in genes *ABCB1* (7q21.12) and *ABCB4* (7q21.12) in the group with a good response was 17% (15/88 cases) versus 2.4% (1/41 cases) in the other group (Fisher’s exact test, *p* = 0.01). The same result was shown for *ABCB8*; this gene was deleted in patients with complete and partial regression in 19.3% of cases (17/88 patients) versus 2.4% (1/41 patients) in patients with stabilization and progression (Fisher’s exact test, *p* = 0.02). Interestingly, for the *ABCB10* gene, a high frequency of amplifications (69.3%, 61/88 cases) was associated with a good response to treatment, whereas in the group with no response, the frequency of amplifications was twice as low (36.6%, 15/41 cases), with *p* = 0.0003 (Table 2).

Significant differences in the frequency of chromosomal aberrations in the *ABCC* family were established for only three genes: *ABCC7, ABCC11,* and *ABCC12.* In all cases, the frequency of deletions in the group with an objective response was statistically significantly higher than in the other group. Moreover, for the genes *ABCC11* and *ABCC12,* the frequency of deletions exceeded 50%. For *ABCC7*, it was 20.5% (18/88 cases) versus 4.9% (2/41 cases) (*p* = 0.03); for *ABCC11* and *ABCC12,* it was 51.1% (45/88 cases; *p* = 0.04). For genes *ABCF2* and *ABCG4*, deletion was also associated with a favourable response to chemotherapy. The frequency of *ABCF2* and *ABCG4* gene deletions was 19.3 (17/88) and 53.4% (47/88), versus 2.4 and 34.1% in the without-answer group (*p* = 0.02 for *ABCF2* and *p* = 0.05 for *ABCG4*).

**Figure 1:**
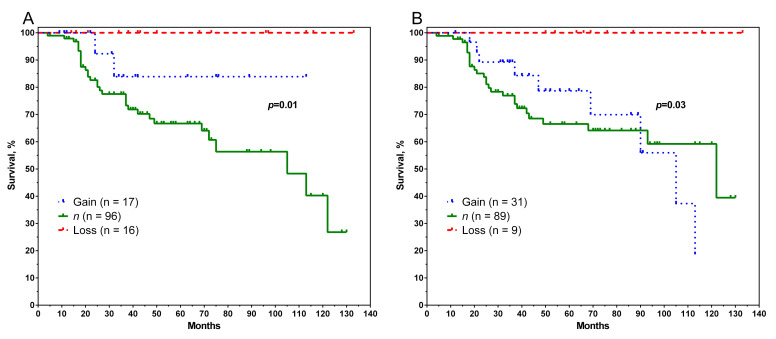
Curves of metastatic-free survival of breast cancer patients depending on the presence of CNA in genes *ABCB1* and *ABCB4* (**A**), as well as *ABCC1* and *ABCC6* (**B**). Note: Gain—amplification; Loss—deletion; *n*—normal number of gene copies.

The highest metastatic survival rates were achieved in patients with deletions in the genes *ABCB1, ABCB4, ABCC1,* and *ABCC6. ABCB1* and *ABCB4* are on the same chromosome locus (7q21.12), as are *ABCC1* and *ABCC6* (16p13.11), and the graphs of metastatic survival are the same for each of these two pairs of genes (Figure 1).

Patients with gene deletions had 100% metastatic-free survival compared to patients with normal gene copies. The 10-year metastatic-free survival rates in those patients with gene deletions were 40 and 39% for *ABCB1*/*4* (Figure 1A) and *ABCC1*/6 (Figure 1B), respectively, and the differences were statistically significant (log-rank test *p* = 0.01 and *p* = 0.03). Other genes investigated showed no association with metastasis-free survival.

### 3.2. Assessment of Transcript Levels of ABC Transporter Genes

In addition, for 39 patients with the luminal B subtype of breast cancer, the expression of 49 studied genes of the ABC transporter family was assessed using a microarray before and after NAC. We also analysed the relationship between gene expression and the main clinical and pathological characteristics of patients. No statistically significant differences were found (data not shown).

An interesting result was shown for the chemotherapy effect. Table S2 (Supplementary Materials) presents the data relationship between the initial expression level and the effectiveness of neoadjuvant chemotherapy. All patients with an objective NAC response had significantly higher levels of *ABCB1, ABCB2, ABCB3, ABCB7, ABCC5, ABCF1,* and *ABCF3* gene expression in the tumour before treatment than in the group with no response (Supplementary Materials, Table S2). However, Bonferroni’s correction for multiple comparisons did not show statistical significance for any gene. It is important to note that the result obtained is consistent with the results of our previous studies, as well as the data available in the literature; the initial level of gene expression is weakly associated with the effectiveness of NAC in breast cancer [2,28,29]. However, the change in expression (increase/decrease) during treatment determines the response to chemotherapy [2].

Based on this, we compared the expression levels before and after NAC in patients with different responses to neoadjuvant chemotherapy. In the group of patients with an objective response to chemotherapy (complete and partial regression), the expression of 28/49 genes (57%) decreased statistically significantly (with 6 × 10^−5^ < *p* < 0.04). This group includes the following genes: *ABCA2, ABCA3, ABCA5, ABCA6, ABCA7, ABCA11P, ABCA12, ABCB1, ABCB2, ABCB3, ABCB4, ABCB6, ABCB7, ABCB11, ABCC1, ABCC6, ABCC9, ABCC10, ABCC11, ABCC12, ABCD2, ABCE1, ABCF1, ABCG1, ABCG2, ABCG4, ABCG5,* and *ABCG8* (Table 3). A statistically significant increase was observed in 9/49 (18%) genes, and expression did not change in 12/49 (25%) genes. Patients with stabilization and tumour progression had increased expression of most of the studied ABC genes. In total, expression increased in 32/49 genes (65%) and significantly increased in 17/49 genes (35%, with 6 × 10^−4^ < *p* < 0.01): *ABCA4, ABCA6, ABCA8, ABCA13, ABCB1, ABCB11, ABCC1, ABCC3, ABCC7, ABCC8, ABCC11, ABCC12, ABCG1, ABCG2, ABCG4, ABCG5,* and *ABCG8*. A comparison of the frequencies of increased and decreased gene expression in patients with different responses to NAC showed statistically significant differences according to the chi-square test (chi-square = 14.83, *p* = 0.0002). It is important to note that the gene expression levels of *ABCA6, ABCB1, ABCB11, ABCC1, ABCC11, ABCC12, ABCG1, ABCG2, ABCG4, ABCG5,* and *ABCG8* significantly changed and were associated with the response to NAC in both groups of patients (Table 3).

Next, using the Kaplan–Meier method, we evaluated the relationships between metastatic-free survival and the expression levels of ABC genes. Distant metastases developed in 16 (41%) patients within 4–130 months from the date of diagnosis. The 1-year metastatic-free survival rate was 94.9%, the 2-year rate was 75.0%, and the 5-year rate was 58.3%.

**Figure 2:**
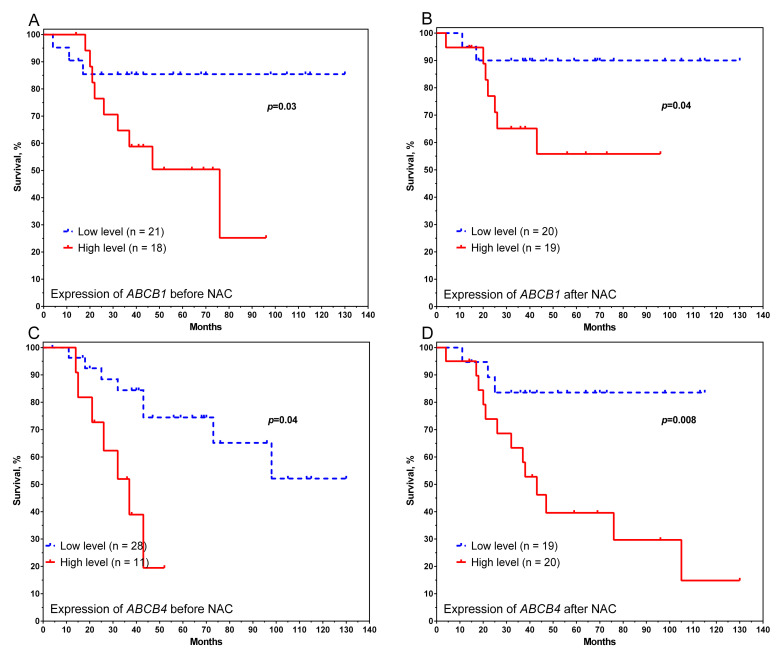
Curves of metastatic survival of patients with breast cancer, depending on the level of *ABCB1* gene expression before (**A**) and after (**B**), and *ABCB4* before (**C**) and after (**D**). Note: the median expression was used as a grouping variable; for *ABCB1* before treatment, 6.06; for *ABCB1* after NAC, 7.4; for *ABCB4* before treatment, 6.62; for *ABCB4* after NAC, 9.46.

We used the median gene expression rates to divide the patients into high-expression and low-expression groups and identify the relationship between expression rates and survival for equal numbers of patients in the two groups. Accordingly, the group with a high level of expression consisted of patients with gene expression more than the median value, and the group with a low level of expression consisted of patients with gene expression below the median value. As a result, it was found that a low level of *ABCB1* and *ABCB4* gene expression before and after NAC was a favourable prognostic factor (Figure 2).

In particular, the 5-year survival rate for patients with high *ABCB1* gene expression before treatment were 50% versus 86% in the other group (log-rank test *p* = 0.03) (Figure 2A). A similar result was observed in the surgically removed tissue: a high level of expression was associated with a low survival rate of breast cancer patients (55% in the group with high expression of *ABCB1* versus 90% in the group with low expression, with a log-rank test *p* = 0.04) (Figure 2B). A similar result was shown for the *ABCB4* gene. The 5-year survival rates for patients with high expression levels before and after treatment were low, at 38% and 40%, respectively, and the 5-year survival rate for patients with low expression levels was 84% (log-rank test *p* = 0.04 and *p* = 0.008 for *ABCB4* before and after treatment, respectively) (Figure 2C,D).

**Figure 3:**
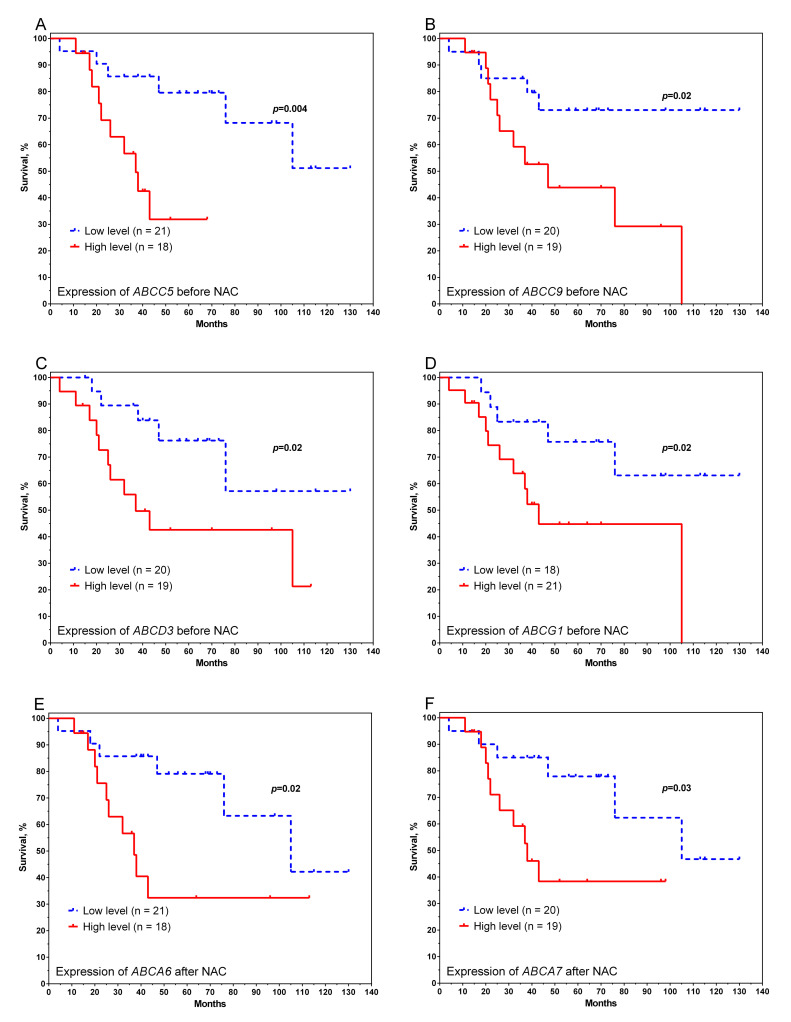
Curves of metastatic survival of breast cancer patients depending on the initial level of expression of genes *ABCC5* (**A**), *ABCC9* (**B**), *ABCD3* (**C**), and *ABCG1* (**D**), as well as the postoperative level of expression of genes *ABCA6* (**E**) and *ABCA7* (**F**). Note: the median of expression was used as a grouping variable: for *ABCC5*, 8.28; *ABCC9,* 7.46; *ABCD3*, 6.73; *ABCG1*, 8.32; *ABCA6*, 6.35; *ABCA7*, 8.54.

Low initial expression levels of the genes *ABCC5, ABCC9, ABCD3*, and *ABCG1* statistically significantly contributed to high survival rates (Figure 3A–D). The 5-year metastatic-free survival rate was 80% for *ABCC5*, 73% for *ABCC9*, 76% for *ABCD3*, and 76% for *ABCG1* in the low-gene-expression group and 32% for *ABCC5*, 44% for *ABCC9*, 43% for *ABCD3*, and 45% for *ABCG1* in the high-gene-expression group (log-rank test *p* = 0.004, *p* = 0.02, *p* = 0.02, *p* = 0.02, respectively).

The relationship between postoperative expression level and disease prognosis is shown for two genes of the *ABCA* family (Figure 3E,F). High expression levels of *ABCA6* and *ABCA7* were associated with low rates of metastasis-free survival (32% and 39%, respectively), whereas the 5-year survival rates for patients with low expression levels were 79% and 78%, respectively (log-rank test *p* = 0.02 and *p* = 0.03). The relationship between expression and metastatic survival is not shown for other studied ABC transporter genes.

### 3.3. Gene Expression Clustering of ABC Transporter

**Figure 4:**
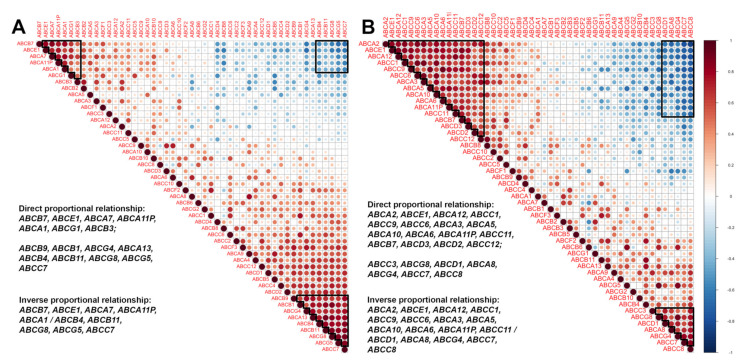
Correlation matrix ABC transporter gene expression before (**A**) and after (**B**) treatment in patients with objective response. Note: groups of coexpression genes marked with a black line (Spearmen coefficient, r > 0.5 or rs < −0.5, *p* ≤ 0.05).

There is an assumption that the formation of the multiple drug resistance phenotype occurs due to the expression of a group of ABC genes (not single genes) [30,31]. Based on that, we assumed that ABC genes have the ability to make a functional cluster. This cluster has a common complex mechanism of expression regulation and the formation of a drug resistance phenotype due to the work of a group of ABC transporter genes and may be associated with the response of a breast tumour to neoadjuvant chemotherapy. In most patients, such a cluster causes a unidirectional change in gene expression in a tumour under the influence of chemotherapy. First, this should be studied by determining the coexpression of the studied ABC genes (Figure 4).

**Figure 5:**
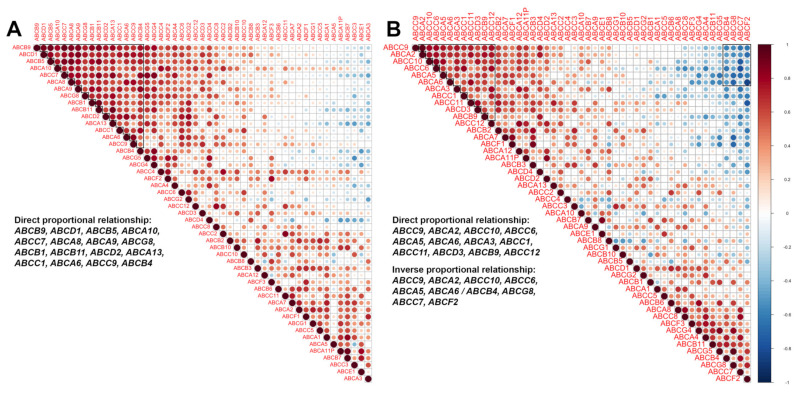
Correlation matrix of ABC transporter gene expression before (**A**) and after (**B**) treatment in patients without response. Note: groups of coexpression genes marked with black line (Spearmen coefficient, r > 0.5 or rs < −0.5, *p* ≤ 0.05).

A correlation analysis of the expression of all ABC transporter genes was carried out. Based on the analysis performed and the resulting Spearman correlation coefficient, a correlation matrix was built (Figure 4 and Figure 5). A matrix was built for gene expression before and after neoadjuvant chemotherapy, depending on the response to treatment. Clustering of the ABC genes was determined automatically by choosing the highest value of the Spearman correlation coefficient.

Figure 4A shows the correlation matrix of ABC transporter gene expression in patients with an objective response to treatment. Three groups of genes formed in these patients before neoadjuvant treatment, two of which had a direct correlation: *ABCB7, ABCE1, ABCA7, ABCA11P, ABCA1, ABCG1, ABCB3* and *ABCB9, ABCB1, ABCG4, ABCA13, ABCB4, ABCB11, ABCG8, ABCG5*, and *ABCC7*. These groups of genes form expression clusters in which coexpression is observed. One groups of genes had inverse correlation: *ABCB7, ABCE1, ABCA7, ABCA11P, ABCA1/ABCB4, ABCB11, ABCG8, ABCG5,* and *ABCC7* (0.5 ≥ rs ≥ −0.5, *p* ≤ 0.05). In this case, high expression of some genes (*ABCB7, ABCE1, ABCA7, ABCA11P,* and *ABCA1*) was associated with low expression of other genes (*ABCG1, ABCB4, ABCB11, ABCG8, ABCG5,* and *ABCC7*).

However, the number of clustered genes after chemotherapy increased (Figure 4B), thus forming a new gene cluster. Two groups of genes also have a direct correlation: *ABCA2, ABCE1, ABCA12, ABCC1, ABCC9, ABCC6, ABCA3, ABCA5, ABCA10, ABCA6, ABCA11P, ABCC11, ABCB7, ABCD3, ABCD2,* and *ABCC12* and the group comprising *ABCC3, ABCG8, ABCD1, ABCA8, ABCG4, ABCC7,* and *ABCC8.* One group of genes had an inverse correlation: *ABCA2, ABCE1, ABCA12, ABCC1, ABCC9, ABCC6, ABCA3, ABCA5, ABCA10, ABCA6, ABCA11P, ABCC11/ABCD1, ABCA8, ABCG4, ABCC7,* and *ABCC8.*

Interestingly, patients who did not respond to therapy had a cluster of 15 genes in biopsy samples: *ABCB9, ABCD1, ABCB5, ABCA10, ABCC7, ABCA8, ABCA9, ABCG8, ABCB1, ABCB11, ABCD2, ABCA13, ABCC1, ABCA6, ABCC9,* and *ABCB4* (Figure 5A).

Groups of coexpressed genes are marked with a black line (Spearman coefficient, r > 0.5 or rs < −0.5, *p* ≤ 0.05).

The number of genes with direct proportional relationships of coexpression was low after treatment (*ABCC9, ABCA2, ABCC10, ABCC6, ABCA5, ABCA6, ABCA3, ABCC1, ABCC11, ABCD3, ABCB9,* and *ABCC12*), but a new group with an inverse proportion appeared: *ABCC9, ABCA2, ABCC10, ABCC6, ABCA5, ABCA6/ABCB4, ABCG8, ABCC7,* and *ABCF2* (Figure 5B).

Thus, there are common ABC gene expression profiles in breast cancer. The presence of these clusters may play a key role in tumour drug resistance. The relationship between the ABC gene expression profiles and some clinical and pathological features also indicates a wide range of functions of these genes that is not limited to the transport of chemotherapy drugs. However, the main limitation was the number of patients, so further studies with a larger sample of patients are needed.

## 4. Discussion

Tumour cells develop chemoresistance due to the overexpression of ABC transporter genes in breast cancer patients. In particular, the genes *ABCA2, ABCA3, ABCB1, ABCB4, ABCC1, ABCC2, ABCC4, ABCC5, ABCC10, ABCC11*, *ABCG1*, and *ABCG2* are directly related to tumour chemoresistance [24,28]. In a recent study, breast cancer cell lines MCF-7 and MDA-MB-231 showed increased copy numbers of *ABCB1, ABCB4* (MCF-7 model), and *ABCA9* (MDA-MB-231 model) to be associated with the formation of docetaxel resistance [32]. Previous studies by the same authors confirmed that *ABCB1* mRNA activation is the main mechanism of breast tumour resistance to docetaxel in in vitro models [33]. It was also shown in oesophageal cancer that the presence of 7q21.12 amplification, where the *ABCB1* gene is located, is associated with the resistance of tumour cells to taxanes [34]. ABCB4 is a phospholipid floppase expressed mainly in the canalicular membrane of human hepatocytes and involved in the transport of phospholipids from liver hepatocytes to bile. Alternative splicing of this gene results in several products of undetermined function. It is believed that the *ABCB4* gene is not involved in the formation of MDR [35]; nevertheless, our study showed the effect of the expression and CNA of this gene on the outcome of chemotherapy and survival. Perhaps this can be explained by the fact that ABCB4 is an important paralogue of the ABCB1 gene [36]. Despite the fact that there are studies of the relationship between the clinical and pathological characteristics of breast cancer patients with CNA status [37,38,39], there is little work on the prognostic value of CNA status. A recent study by R. Kikuchi-Koike et al. (2019) showed that deletion of the presence or normal CNA status of *ABCA3* is a potential indicator of high-risk relapse in breast cancer patients [40]. Interestingly, although the association between the expression of *ABCB1, ABCC1, ABCC2, ABCG1, ABCG2*, and other genes and the effect of NAC has been shown in many studies [41,42], for the genes of the *ABCA* subfamily, the association with the effect of NAC is not very obvious. However, high expression of *ABCA2* and *ABCA3* was correlated with poor chemotherapy response [43]. An early study also indicated that decreased *ABCA3* expression is an independent adverse risk factor for breast tumour recurrence (log-rank test *p* = 0.0053) [44]. At the same time, *ABCA1* expression was not associated with disease-free survival rates (log-rank test *p* = 0.0587). Notably, the expression levels of *ABCA9, ABCA10*, and *ABCC9* were associated with disease-free survival [45].

The role of the *ABCF1* gene remains unclear, as it does not have a transmembrane domain and its function differs from that of most ABC transporters [46]. Nevertheless, we showed that decreased *ABCF1* gene expression in breast tumours is associated with chemosensitivity. A similar result was shown in hepatocellular carcinoma [47]. Hyperexpression of the *ABCB1* and *ABCB4* genes before and after treatment was associated with the NAC effect and low rates of metastasis-free survival. This explains, to some extent, the results reported by Huang et al., 2018 who found that overexpression of the *ABCB4* gene in breast cancer cell lines is associated with resistance to doxorubicin and, as a consequence, may determine the tumour response to chemotherapy [48]. The association of the *ABCB1* gene with prognosis has been shown for polymorphisms of this gene [49].

There are only a few studies looking at the expression cluster of ABC transporter genes [50,51,52]. However, it is important to note that the formation of the drug resistance phenotype is due to the work of several main ABC genes [53]. The study of multidrug resistance is basically reduced to considering the relationship of individual genes, such as *ABCB1*, *ABCC1*, and *ABCG2*, with various clinical and morphological parameters, the effectiveness of chemotherapy, and the outcome of the disease [54]. Nevertheless, the genes *ABCA3, ABCA8, ABCA12, ABCC7*, and *ABCC8* cluster in breast tumours, and the expression of this gene group was associated with the clinical and pathological parameters of tumours [55]. In another study, ABC genes were distributed across clusters, which allowed us to determine gene groups with increased and decreased expression and connections with relapse-free survival [56]. In addition, some authors note that the coexpression of P-glycoprotein (product of the *ABCB1* gene), *ABCG2*, and *ABCC1* proteins in breast tumours before treatment and after NAC was not detected [29]. Positive pairwise correlations of the ABC gene expression level were shown, and a significant level of correlation was found for the *ABCB1/ABCG2*, *ABCB1/ABCC1*, and *MVP/ABCC1* gene pairs [57].

In addition, some authors have evaluated the genetic variability of ABC transporters in breast cancer. The *ABCC1* gene polymorphism (rs45511401) is a predictor of disease-specific survival. This association is highly drug-specific for subgroups treated with the *ABCC1* substrates cyclophosphamide (log-rank *p* = 0.0011) and doxorubicin (log-rank *p* = 0.0088), independent of age and tumour stage, whereas no association was found in individuals treated with tamoxifen (log-rank *p* = 0.13) [58]. In the same year, a similar study was conducted in which the authors showed the role of genetic variability of ABC transporters in the prognosis of breast cancer and response to chemotherapy. *ABCA4* (rs2275032), *ABCA9* (rs11871944), *ABCA12* (rs71428357), *ABCB5* (rs3210441), *ABCC5* (rs4148579), *ABCC8* (rs739689), *ABCC11* (rs17822931), and *ABCD4* (rs2301347 and rs23013) gene variants are associated with chemotherapy response. Polymorphisms of the *ABCA7* (rs9282562), *ABCA13* (rs17548783 and rs74859514), *ABCC4* (rs899494), and *ABCG8* (rs34198326) genes were correlated with relapse-free survival of patients, and the presence of a rare homozygous genotype is associated with lower survival rates, with log-rank *p* < 0.05 (except *ABCG8* (rs34198326)) [59]. Similar associations were not detected for *ABCB1* (log-rank *p* = 0.08) or *ABCG2* (log-rank *p* = 0.061). A 2018 meta-analysis showed that the polymorphism of the *ABCB1* rs1045642 (C > T) gene was statistically significantly associated with poor progression-free survival (HR = 1.33, 95% CI: 1.07–1.64, *p* = 0.046). The relationship between *ABCC13* (rs2205253) and overall survival rates was also established (HR = 0.49, 95% CI: 0.33–0.71, *p* = 2.12 × 10^−4^) [60]. Despite the fact that polymorphisms of the ABC transporter genes cannot directly affect the effectiveness of chemotherapy and the survival of patients with breast cancer, genetic variability can affect the positive and negative regulation of the activity of these genes.

Thus, in this study, the aberrant state of some ABC transporter genes and a decrease in their expression during neoadjuvant chemotherapy were shown to be predictors of the treatment effectiveness and potential prognostic markers of metastatic survival.

## Figures and Tables

**Table 1:** Clinical and pathological parameters of breast cancer patients.

Clinical and Pathological Parameter		Number of Patients, abs. (%)
Age	≤45	54 (41.9)
	>45	75 (58.1)
Menstrual status	Perimenopause	65 (50.4)
	Postmenopause	64 (49.6)
Histological type	Invasive ductal cancer	93 (72.1)
	Invasive lobular carcinoma	36 (27.9)
Size	T_1_	18 (14.0)
	T_2_	95 (73.6)
	T_3_	7 (5.4)
	T_4_	9 (7.0)
Lymphogenous metastasis	N_0_	54 (41.9)
	N_1_	55 (42.6)
	N_2_	9 (7.0)
	N_3_	11 (8.5)
Histological form	Unicentric	57 (44.2)
	Multicentric	72 (55.8)
Molecular subtype	Luminal B HER2-negative	95 (73.6)
	HER2+	12 (9.3)
	Triple negative	22 (17.1)
NAC scheme	CAX	28 (21.7)
	AC	44 (34.1)
	Taxotere	26 (20.2)
	AT/ACT	16 (12.4)
	CP	15 (11.6)
NAC effect	Complete regression	13 (10.1)
	Partial regression	75 (58.1)
	Stabilization	32 (24.8)
	Progression	9 (7.0)

**Table 2:** Frequency of chromosomal aberration ABC transporter genes in breast tumours in patients with different responses to NAC.

Genes	CR + PR (*n* = 88)			P + ST (*n* = 41)			*p*-Level
	Loss	*n*	Gain	Loss	*n*	Gain	
*ABCA1*	20 (22.7)	63 (71.6)	5 (5.7)	9 (22.0)	29 (70.7)	3 (7.3)	0.93
*ABCA2*	19 (21.6)	59 (67.0)	10 (11.4)	(17.1)	28 (68.3)	6 (14.6)	0.76
*ABCA3*	9 (10.2)	50 (56.8)	29 (33.0)	4 (9.8)	28 (68.3)	9 (22.0)	0.41
*ABCA4*	25 (28.4)	57 (64.8)	6 (6.8)	10 (24.4)	27 (65.9)	4 (9.8)	0.78
*ABCA5*	14 (15.9)	45 (51.1)	29 (33.0)	6 (14.6)	22 (53.7)	13 (31.7)	0.96
*ABCA6*	10 (11.4)	43 (48.9)	35 (39.8)	6 (14.6)	20 (48.8)	15 (36.6)	0.85
*ABCA7*	27 (30.7)	60 (68.2)	1 (1.1)	13 (31.7)	28 (68.3)	0 (0.0)	0.78
*ABCA8*	10 (11.4)	43 (48.9)	35 (39.8)	6 (14.6)	20 (48.8)	15 (36.6)	0.85
*ABCA9*	10 (11.4)	43 (48.9)	35 (39.8)	6 (14.6)	20 (48.8)	15 (36.6)	0.85
*ABCA10*	14 (15.9)	45 (51.1)	29 (33.0)	6 (14.6)	22 (53.7)	13 (31.7)	0.96
*ABCA11P*	26 (29.5)	56 (63.6)	6 (6.8)	13 (31.7)	25 (61.0)	3 (7.3)	0.96
*ABCA12*	17 (19.3)	66 (75.0)	5 (5.7)	13 (31.7)	28 (68.3	0 (0.0)	0.11
*ABCA13*	6 (6.8)	67 (76.1)	15 (17.0)	2 (4.9)	34 (82.9)	5 (12.2)	0.68
*ABCB1*	**15 (17.0)**	**61 (69.3)**	12 (13.6)	**1 (2.4)**	**35 (85.4)**	5 (12.2)	**0.01**
*ABCB2*	14 (15.9)	65 (73.9)	9 (10.2)	2 (4.9)	33 (80.5)	6 (14.6)	0.18
*ABCB3*	14 (15.9)	65 (73.9)	9 (10.2)	2 (4.9)	33 (80.5)	6 (14.6)	0.18
*ABCB4*	**15 (17.)**	**61 (69.3)**	12 (13.6)	**1 (2.4)**	**35 (85.4)**	5 (12.2)	**0.01**
*ABCB5*	7 (8.0)	66 (75.0)	15 (17.0)	2 (4.9)	31 (75.6)	8 (19.5)	0.78
*ABCB6*	**17 (19.3)**	**66 (75.0)**	**5 (5.7)**	**13 (31.7)**	**28 (68.3)**	**0 (0.0)**	**0.01**
*ABCB7*	16 (18.2)	70 (79.5)	2 (2.3)	7 (17.1)	29 (70.7)	5 (12.2)	0.06
*ABCB8*	**17 (19.3)**	**58 (65.9)**	13 (14.8)	**1 (2.4)**	**31 (75.6)**	9 (22.0)	**0.02**
*ABCB9*	16 (18.2)	61 (69.3)	11 (12.5)	5 (12.2)	32 (78.0)	4 (9.8)	0.57
*ABCB10*	4 (4.5)	**23 (26.1)**	**61 (69.3)**	2 (4.9)	**24 (58.5)**	**15 (36.6)**	**3 × 10 ^−4^**
*ABCB11*	18 (20.5)	63 (71.6)	7 (8.0)	10 (24.4)	30 (73.2)	1 (2.4)	0.45
*ABCC1*	7 (8.0)	57 (64.8)	24 (27.3)	2 (4.9)	32 (78.0)	7 (17.1)	0.31
*ABCC2*	29 (33.0)	56 (63.6)	3 (3.4)	12 (29.3)	28 (68.3)	1 (2.4)	0.86
*ABCC3*	17 (19.3)	42 (47.7)	29 (33.0)	11 (26.8)	19 (46.3)	11 (26.8)	0.58
*ABCC4*	32 (36.4)	46 (52.3)	10 (11.4)	11 (26.8)	27 (65.9)	3 (7.3)	0.34
*ABCC5*	8 (9.1)	62 (70.5)	18 (20.5)	6 (14.6)	28 (68.3)	7 (17.1)	0.61
*ABCC6*	7 (8.0)	57 (64.8)	24 (27.3)	2 (4.9)	32 (78.0)	7 (17.1)	0.31
*ABCC7*	**18 (20.5)**	**62 (70.5)**	8 (9.1)	**2 (4.9)**	**36 (87.8)**	3 (7.3)	**0.03**
*ABCC8*	25 (28.4)	57 (64.8)	6 (6.8)	5 (12.2)	31 (75.6)	5 (12.2)	0.10
*ABCC9*	12 (13.6)	68 (77.3)	8 (9.1)	7 (17.1)	28 (68.3)	6 (14.6)	0.51
*ABCC10*	14 (15.9)	65 (73.9)	9 (10.2)	5 (12.2)	28 (68.3)	8 (19.5)	0.33
*ABCC11*	**45 (51.1)**	**37 (42.0)**	6 (6.8)	**12 (29.3)**	**25 (61.0)**	4 (9.8)	**0.04**
*ABCC12*	**45 (51.1)**	**37 (42.0)**	6 (6.8)	**12 (29.3)**	**25 (61.0)**	4 (9.8)	**0.04**
*ABCD1*	16 (18.2)	65 (73.9)	7 (8.0)	5 (12.2)	29 (70.7)	7 (17.1)	0.24
*ABCD2*	12 (13.6)	66 (75.0)	10 (11.4)	3 (7.3)	33 (80.5)	5 (12.2)	0.57
*ABCD3*	23 (26.1)	59 (67.0)	6 (6.8)	8 (19.5)	27 (65.9)	6 (14.6)	0.30
*ABCD4*	37 (42.0)	49 (55.7)	2 (2.3)	14 (34.1)	27 (65.9)	0 (0.0)	0.39
*ABCE1*	20 (22.7)	64 (72.7)	4 (4.5)	10 (24.4)	30 (73.2)	1 (2.4)	0.83
*ABCF1*	14 (15.9)	65 (73.9)	9 (10.2)	2 (4.9)	33 (80.5)	6 (14.6)	0.18
*ABCF2*	**17 (19.3)**	**58 (65.9)**	13 (14.8)	**1 (2.4)**	**31 (75.6)**	9 (22.0)	**0.02**
*ABCF3*	8 (9.1)	62 (70.5)	18 (20.5)	6 (14.6	28 (68.3)	7 (17.1)	0.39
*ABCG1*	14 (15.9)	61 (69.3)	13 (14.8)	7 (17.1	31 (75.6)	3 (7.3)	0.48
*ABCG2*	22 (25.0)	61 (69.3)	5 (5.7)	10 (24.4)	29 (70.7)	2 (4.9)	0.97
*ABCG4*	**47 (53.4)**	**38 (43.2)**	3 (3.4)	**14 (34.1)**	**25 (61.0)**	2 (4.9)	**0.05**
*ABCG5*	17 (19.3)	63 (71.6)	8 (9.1)	7 (17.1)	30 (73.2)	4 (9.8)	0.95
*ABCG8*	17 (19.3)	63 (71.6)	8 (9.1)	7 (17.1)	30 (73.2)	4 (9.8)	0.95

Note: CR + PR—complete and partial regression, P + ST—progression and stabilization. Statistically significant differences are shown in bold. Note: Gain—amplification; Loss—deletion; *n*—normal number of gene copies.

**Table 3:** Expression of ABC transporters in patients with breast cancer before and after neoadjuvant treatment depending on NAC effect.

Gene	CR + PR (*n* = 25)			*p*-Level	P + ST (*n* = 14)			*p*-Level
	Before	After	Change		Before	After	Change	
*ABCA1*	10.03 ± 0.32	9.47 ± 0.25	Decrease	0.11	9.34 ± 0.43	8.79 ± 0.39	Decrease	0.42
*ABCA2*	***7.29 ± 0.17***	***5.52 ± 0.29***	***Decrease***	***1 × 10^−3^***	7.43 ± 0.18	5.25 ± 0.37	Decrease	3 × 10^−3^
*ABCA3*	***7.94 ± 0.25***	***6.10 ± 0.33***	***Decrease***	***5 × 10^−3^***	7.83 ± 0.29	6.43 ± 0.40	Decrease	0.18
*ABCA4*	6.84 ± 0.17	9.52 ± 0.49	Increase	1 × 10^−3^	**6.36 ± 0.33**	**9.54 ± 0.66**	**Increase**	**0.01**
*ABCA5*	***9.89 ± 0.28***	***6.45 ± 0.43***	***Decrease***	***6 × 10^−5^***	9.52 ± 0.42	5.92 ± 0.45	Decrease	0.01
*ABCA6*	**7.32 ± 0.18**	**6.78 ± 0.22**	**Decrease**	**0.04**	**7.30 ± 0.25**	**9.14 ± 0.21**	**Increase**	**0.01**
*ABCA7*	***9.63 ± 0.32***	***8.59 ± 0.24***	***Decrease***	***2 × 10^−3^***	8.93 ± 0.39	7.98 ± 0.58	Decrease	0.18
*ABCA8*	7.93 ± 0.29	11.41 ± 0.36	Increase	1 × 10^−5^	7.90 ± 0.35	10.61 ± 0.64	Increase	0.01
*ABCA9*	7.51 ± 0.23	8.97 ± 0.23	Increase	5 × 10^−3^	7.34 ± 0.29	7.81 ± 0.35	Increase	0.78
*ABCA10*	6.52 ± 0.25	6.35 ± 0.41	Decrease	0.68	5.86 ± 0.26	6.15 ± 0.39	Increase	0.42
*ABCA11P*	***8.71 ± 0.29***	***5.72 ± 0.36***	***Decrease***	***6 × 10^−5^***	7.92 ± 0.40	6.55 ± 0.52	Decrease	0.18
*ABCA12*	***9.84 ± 0.44***	***7.16 ± 0.33***	***Decrease***	***3 × 10^−4^***	8.79 ± 0.77	7.08 ± 0.31	Decrease	0.42
*ABCA13*	6.75 ± 0.25	7.94 ± 0.25	Increase	5 × 10^−3^	**6.57 ± 0.34**	**7.77 ± 0.41**	**Increase**	**0.01**
*ABCB1*	***6.39 ± 0.19***	***4.32 ± 0.23***	***Decrease***	***5 × 10^−4^***	***5.87 ± 0.27***	***9.35 ± 0.55***	***Increase***	***6 × 10^−4^***
*ABCB2*	**10.59 ± 0.32**	**9.98 ± 0.29**	**Decrease**	**0.04**	9.76 ± 0.36	9.07 ± 0.58	Decrease	0.18
*ABCB3*	***8.73 ± 0.23***	***8.25 ± 0.26***	***Decrease***	***5 × 10^−3^***	7.68 ± 0.33	7.84 ± 0.46	Increase	0.78
*ABCB4*	***6.80 ± 0.24***	***4.71 ± 0.36***	***Decrease***	***3 × 10^−4^***	6.78 ± 0.32	8.75 ± 0.41	Increase	0.18
*ABCB5*	7.21 ± 0.38	8.35 ± 0.25	Increase	0.11	6.68 ± 0.49	7.49 ± 0.31	Increase	0.42
*ABCB6*	**5.73 ± 0.15**	**5.56 ± 0.40**	**Decrease**	**0.01**	5.43 ± 0.23	7.01 ± 0.53	Increase	0.18
*ABCB7*	**9.84 ± 0.30**	**7.45 ± 0.37**	**Decrease**	**0.01**	8.64 ± 0.46	7.39 ± 0.40	Decrease	0.42
*ABCB8*	6.36 ± 0.13	6.51 ± 0.27	Increase	1.00	5.98 ± 0.26	6.66 ± 0.44	Increase	0.78
*ABCB9*	6.52 ± 0.21	6.15 ± 0.13	Decrease	1.00	6.22 ± 0.22	5.93 ± 0.22	Decrease	0.78
*ABCB10*	8.38 ± 0.18	8.22 ± 0.16	Decrease	0.42	7.89 ± 0.38	7.95 ± 0.22	Increase	0.78
*ABCB11*	***5.78 ± 0.25***	***3.61 ± 0.29***	***Decrease***	***6 × 10^−5^***	**5.64 ± 0.35**	**7.70 ± 0.35**	**Increase**	**0.01**
*ABCC1*	***8.61 ± 0.27***	***7.17 ± 0.26***	***Decrease***	***5 × 10^−3^***	**8.71 ± 0.34**	**8.96 ± 0.67**	**Increase**	**0.01**
*ABCC2*	5.18 ± 0.21	5.16 ± 0.19	Decrease	0.83	5.54 ± 0.59	5.95 ± 0.26	Increase	0.78
*ABCC3*	9.13 ± 0.44	10.23 ± 0.31	Increase	0.11	**7.87 ± 0.37**	**8.93 ± 0.55**	**Increase**	**0.05**
*ABCC4*	7.45 ± 0.17	7.24 ± 0.17	Decrease	1.00	7.44 ± 0.37	7.50 ± 0.17	Increase	0.78
*ABCC5*	8.58 ± 0.23	7.90 ± 0.16	Decrease	0.31	7.72 ± 0.34	7.46 ± 0.31	Decrease	0.78
*ABCC6*	***8.42 ± 0.17***	***6.48 ± 0.31***	***Decrease***	***1 × 10^−3^***	8.22 ± 0.29	6.67 ± 0.40	Decrease	0.06
*ABCC7*	5.64 ± 0.20	9.19 ± 0.47	Increase	6 × 10^−5^	**5.31 ± 0.30**	**8.63 ± 0.50**	**Increase**	**0.01**
*ABCC8*	6.10 ± 0.16	9.11 ± 0.43	Increase	1 × 10^−3^	***5.78 ± 0.19***	***8.16 ± 0.52***	***Increase***	***3 × 10^−3^***
*ABCC9*	***7.79 ± 0.25***	***6.46 ± 0.42***	***Decrease***	***5 × 10^−3^***	7.42 ± 0.46	5.90 ± 0.41	Decrease	3 × 10^−3^
*ABCC10*	***8.43 ± 0.20***	***6.93 ± 0.20***	***Decrease***	***1 × 10^−5^***	8.24 ± 0.27	8.92 ± 0.44	Increase	0.18
*ABCC11*	**8.58 ± 0.30**	**7.38 ± 0.25**	**Decrease**	**0.01**	***8.18 ± 0.64***	***9.97 ± 0.37***	***Increase***	***1 × 10^−3^***
*ABCC12*	**7.32 ± 0.17**	**6.44 ± 0.29**	**Decrease**	**0.04**	***7.76 ± 0.42***	***9.92 ± 0.44***	***Increase***	***1 × 10^−3^***
*ABCD1*	7.11 ± 0.17	9.55 ± 0.36	Increase	3 × 10^−4^	7.21 ± 0.27	8.31 ± 0.40	Increase	0.18
*ABCD2*	***7.33 ± 0.21***	***6.15 ± 0.24***	***Decrease***	***1 × 10^−3^***	6.81 ± 0.34	6.44 ± 0.46	Decrease	0.57
*ABCD3*	7.60 ± 0.31	7.97 ± 0.19	Increase	0.42	7.17 ± 0.41	8.01 ± 0.44	Increase	0.06
*ABCD4*	6.60 ± 0.16	6.44 ± 0.15	Decrease	0.83	6.29 ± 0.20	6.19 ± 0.17	Decrease	0.78
*ABCE1*	***11.98 ± 0.35***	***9.59 ± 0.42***	***Decrease***	***5 × 10^−3^***	11.39 ± 0.5	9.01 ± 0.44	Decrease	0.06
*ABCF1*	***9.11 ± 0.21***	***8.13 ± 0.32***	***Decrease***	***1 × 10^−3^***	8.50 ± 0.22	7.51 ± 0.33	Decrease	0.06
*ABCF2*	6.95 ± 0.16	8.26 ± 0.20	Increase	6 × 10^−5^	7.00 ± 0.28	8.40 ± 0.31	Increase	0.18
*ABCF3*	6.41 ± 0.13	7.45 ± 0.24	Increase	0.01	5.98 ± 0.19	7.22 ± 0.38	Increase	0.42
*ABCG1*	**8.17 ± 0.29**	**7.98 ± 0.34**	**Decrease**	**0.03**	***7.72 ± 0.41***	***9.08 ± 0.54***	***Increase***	***1 × 10^−3^***
*ABCG2*	***8.78 ± 0.21***	***6.61 ± 0.41***	***Decrease***	***3 × 10^−4^***	**8.91 ± 0.62**	**9.99 ± 0.38**	**Increase**	**0.01**
*ABCG4*	***7.76 ± 0.21***	***5.30 ± 0.41***	***Decrease***	***3 × 10^−4^***	***7.20 ± 0.24***	***9.36 ± 0.39***	***Increase***	***1 × 10^−3^***
*ABCG5*	***7.13 ± 0.20***	***5.02 ± 0.33***	***Decrease***	***3 × 10^−4^***	***7.86 ± 0.24***	***9.90 ± 0.30***	***Increase***	***1 × 10^−3^***
*ABCG8*	***6.86 ± 0.18***	***4.23 ± 0.32***	***Decrease***	***6 × 10^−5^***	***6.83 ± 0.25***	***8.02 ± 0.35***	***Increase***	***1 × 10^−3^***

Note: CR + PR—complete and partial regression, P + ST—progression and stabilization; bold type indicates statistically significant differences in the level of ABC gene expression before and after NAC, depending on the response to treatment. In the first group of patients with an objective response (CR + PR), only a statistically significant decrease in expression was identified; in the group without response to treatment (P + ST), only a statistically significant increase in expression was identified. Statistically significant differences in the level of ABC gene expression before and after NAC, depending on the response to treatment, with Bonferroni correction, are shown in bold and italics. The table shows the mean value of gene expression ± error of the mean (Mean ± SE).

## Data Availability

Database registration certificate RU 2021621350 22/06/2021 Ibragimova, M.K., Tsyganov, M.M., Garbukov, E.Y., Usynin E.A., Litvyakov, N.V. Database of genome-wide expression and copy number gene in breast tumors.

## Supplementary Materials

The following supporting information can be downloaded at: https://www.mdpi.com/article/10.3390/pharmaceutics14050948/s1, Table S1: Frequency of chromosomal aberrations in ABC-transporter genes in breast tumors; Table S2: Expression ABC-transporters in patients with breast cancer before neoadjuvant treatment depending on NAC effect.
