# Supplementary material for: DNA Copy Number Aberrations and Expression of ABC Transporter Genes in Breast Tumour: Correlation with the Effect of Neoadjuvant Chemotherapy and Prognosis of the Disease
Source: Pharmaceutics. 2022 Apr 27;14(5):948. doi: 10.3390/pharmaceutics14050948 (PMC9146568; doi:10.3390/pharmaceutics14050948)
Supplement: Supplementary file 1 [file pharmaceutics-14-00948-s001.zip › Supplement 1 Table S1.pdf]

Supplement 1

Table S1 – Frequency of chromosomal aberrations in ABC-transporter genes in breast tumors.

Genes	Locus chromosome	CNA. abs. n. (%)		
		Loss	No change	Gain
ABCA1	9q31.1	29 (22.5)	92 (71.3)	8 (6.2)
ABCA2	9q34.3	26 (20.2)	87 (67.4)	16 (12.4)
ABCA3	16p13.3	13 (10.1)	78 (60.5)	38 (29.5)
ABCA4	1p22.1	35 (27.1)	84 (65.1)	10 (7.8)
ABCA5	17q24.3	20 (15.5)	67 (51.9)	42 (32.6)
ABCA6	17q24.2	16 (12.4)	63 (48.8)	50 (38.8)
ABCA7	19p13.3	40 (31.0)	88 (68.2)	1 (0.8)
ABCA8	17q24.2	16 (12.4)	63 (48.8)	50 (38.8)
ABCA9	17q24.2	16 (12.4)	63 (48.8)	50 (38.8)
ABCA10	17q24.3	20 (15.5)	67 (51.9)	42 (32.6)
ABCA11P	4p16.3	39 (30.2)	81 (62.8)	9 (7.0)
ABCA12	2q35	30 (23.3)	94 (72.9)	5 (3.9)
ABCA13	7p12.3	8 (6.2)	101 (78.3)	20 (15.5)
ABCB1	7q21.12	16 (12.4)	96 (74.4)	17 (13.2)
ABCB2	6p21.32	16 (12.4)	98 (76.0)	15 (11.6)
ABCB3	6p21.32	16 (12.4)	98 (76.0)	15 (11.6)
ABCB4	7q21.12	16 (12.4)	96 (74.4)	17 (13.2)
ABCB5	7p21.1	9 (7.0)	97 (75.2)	23 (17.8)
ABCB6	2q35	30 (23.3)	94 (72.9)	5 (3.9)
ABCB7	Xq13.3	23 (17.8)	99 (76.7)	7 (5.4)
ABCB8	7q36.1	18 (14.0)	89 (69.0)	22 (17.1)
ABCB9	12q24.31	21 (16.3)	93 (72.1)	15 (11.6)
ABCB10	1q42.13	6 (4.7)	47 (36.4)	76 (58.9)
ABCB11	2q31.1	28 (21.7)	93 (72.1)	8 (6.2)
ABCC1	16p13.11	9 (7.0)	89 (69.0)	31 (24.0)
ABCC2	10q24.2	41 (31.8)	84 (65.1)	4 (3.1)
ABCC3	17q21.33	28 (21.7)	61 (47.3)	40 (31.0)
ABCC4	13q32.1	43 (33.3)	73 (56.6)	13 (10.1)
ABCC5	3q27.1	14 (10.9)	90 (69.8)	25 (19.4)
ABCC6	16p13.11	9 (7.0)	89 (69.0)	31 (24.0)
ABCC7	7q31.2	20 (15.5)	98 (76.0)	11 (8.5)
ABCC8	11p15.1	30 (23.3)	88 (68.2)	11 (8.5)
ABCC9	12p12.1	19 (14.7)	96 (74.4)	14 (10.9)
ABCC10	6p21.1	19 (14.7)	93 (72.1)	17 (13.2)
ABCC11	16q12.1	57 (44.2)	62 (48.1)	10 (7.8)
ABCC12	16q12.1	57 (44.2)	62 (48.1)	10 (7.8)
ABCD1	Xq28	21 (16.3)	94 (72.9)	14 (10.9)
ABCD2	12q12	15 (11.6)	99 (76.7)	15 (11.6)
ABCD3	1p21.3	31 (24.0)	86 (66.7)	12 (9.3)
ABCD4	14q24.3	51 (39.5)	76 (58.9)	2 (1.6)
ABCE1	4q31.21	30 (23.3)	94 (72.9)	5 (3.9)
ABCF1	6p21.33	16 (12.4)	98 (76.0)	15 (11.6)
ABCF2	7q36.1	18 (14.0)	89 (69.0)	22 (17.1)

ABCF3	3q27.1	14 (10.9)	90 (69.8)	25 (19.4)
ABCG1	21q22.3	21 (16.3)	92 (71.3)	16 (12.4)
ABCG2	4q22.1	32 (24.8)	90 (69.8)	7 (5.4)
ABCG4	11q23.3	61 (47.3)	63 (48.8)	5 (3.9)
ABCG5	2p21	24 (18.6)	93 (72.1)	12 (9.3)
ABCG8	2p21	24 (18.6)	93 (72.1)	12 (9.3)

Note: Gain - amplification; Loss - deletion; n - normal number of gene copies (no change)
