# Supplementary material for: DNA Copy Number Aberrations and Expression of ABC Transporter Genes in Breast Tumour: Correlation with the Effect of Neoadjuvant Chemotherapy and Prognosis of the Disease
Source: Pharmaceutics. 2022 Apr 27;14(5):948. doi: 10.3390/pharmaceutics14050948 (PMC9146568; doi:10.3390/pharmaceutics14050948)
Supplement: Supplementary file 1 [file pharmaceutics-14-00948-s001.zip › Supplement 1 Table S2.pdf]

Supplement 1

Table S2 – Expression ABC-transporters in patients with breast cancer before neoadjuvant treatment depending on NAC effect.

Genes	CR+PR (n=25)	P+ST (n=14)	*p*-level
ABCA1	10.03±0.32	9.34±0.43	0.24
ABCA2	7.29±0.17	7.43±0.18	0.68
ABCA3	7.94±0.25	7.83±0.29	0.96
ABCA4	6.84±0.17	6.36±0.33	0.43
ABCA5	9.89±0.28	9.52±0.42	0.84
ABCA6	7.32±0.18	7.30±0.25	0.86
ABCA7	9.63±0.32	8.93±0.39	0.16
ABCA8	7.93±0.29	7.90±0.35	0.91
ABCA9	7.51±0.23	7.34±0.29	0.67
ABCA10	6.52±0.25	5.86±0.26	0.17
ABCA11P	8.71±0.29	7.92±0.40	0.15
ABCA12	9.84±0.44	8.79±0.77	0.09
ABCA13	6.75±0.25	6.57±0.34	0.79
ABCB1	**6.39±0.19**	**5.87±0.27**	**0.04**
ABCB2	**10.59±0.32**	**9.76±0.36**	**0.05**
ABCB3	**8.73±0.23**	**7.68±0.33**	**0.03**
ABCB4	6.80±0.24	6.78±0.32	0.91
ABCB5	7.21±0.38	6.68±0.49	0.46
ABCB6	5.73±0.15	5.43±0.23	0.14
ABCB7	**9.84±0.30**	**8.64±0.46**	**0.04**
ABCB8	6.36±0.13	5.98±0.26	0.18
ABCB9	6.52±0.21	6.22±0.22	0.67
ABCB10	8.38±0.18	7.89±0.38	0.31
ABCB11	5.78±0.25	5.64±0.35	0.83
ABCC1	8.61±0.27	8.71±0.34	0.69
ABCC2	5.18±0.21	5.54±0.59	0.96
ABCC3	9.13±0.44	7.87±0.37	0.06
ABCC4	7.45±0.17	7.44±0.37	0.81
ABCC5	**8.58±0.23**	**7.72±0.34**	**0.04**
ABCC6	8.42±0.17	8.22±0.29	0.56
ABCC7	5.64±0.20	5.31±0.30	0.43
ABCC8	6.10±0.16	5.78±0.19	0.17
ABCC9	7.79±0.25	7.42±0.46	0.28
ABCC10	8.43±0.20	8.24±0.27	0.75
ABCC11	8.58±0.30	8.18±0.64	0.17
ABCC12	7.32±0.17	7.76±0.42	0.54
ABCD1	7.11±0.17	7.21±0.27	0.64
ABCD2	7.33±0.21	6.81±0.34	0.15
ABCD3	7.60±0.31	7.17±0.41	0.62
ABCD4	6.60±0.16	6.29±0.20	0.24
ABCE1	11.98±0.35	11.39±0.46	0.17
ABCF1	**9.11±0.21**	**8.50±0.22**	**0.01**

ABCF2	6.95±0.16	7.00±0.28	0.64
ABCF3	**6.41±0.13**	**5.98±0.19**	**0.05**
ABCG1	8.17±0.29	7.72±0.41	0.30
ABCG2	8.78±0.21	8.91±0.62	0.53
ABCG4	7.76±0.21	7.20±0.24	0.08
ABCG5	7.13±0.20	7.86±0.24	0.46
ABCG8	6.86±0.18	6.83±0.25	0.66

Note: CR+PR - complete and partial regression, P+ST - progression and stabilization. Statistically significant differences are shown in bold. The table shows the mean value of gene expression ± error of the mean (Mean±SE).
